# Hybrid Atrial Fibrillation Ablation: A Decade-Long Single-Center Experience

**DOI:** 10.31083/RCM43780

**Published:** 2025-12-23

**Authors:** Giuseppe Nasso, Walter Vignaroli, Cosimo Domenico Dicandia, Pasquale Filannino, Giuseppe Lembo, Flavio Fiore, Mario Siro Brigiani, Ernesto Greco, Felice Agrò, Giuseppe Santarpino, Giuseppe Speziale

**Affiliations:** ^1^Department of Cardiac Surgery, Anthea Hospital and Santa Maria Hospital GVM Care & Research, 70131 Bari, Italy; ^2^Department of Medicine and Surgery, LUM University, Casamassima, 70010 Bari, Italy; ^3^Department of Cardiac Surgery, San Carlo di Nancy Hospital GVM Care & Research, 00156 Rome, Italy; ^4^Department of Health and Life Sciences European University of Rome, 00163 Rome, Italy; ^5^Anesthesia and Intensive Care Research Unit, Campus Bio-Medico University, 00128 Rome, Italy; ^6^Department of Cardiac Surgery, Città di Lecce Hospital, GVM Care & Research, 73100 Lecce, Italy; ^7^Department of Clinical and Experimental Medicine, Magna Graecia University, 88100 Catanzaro, Italy; ^8^Department of Cardiac Surgery, Paracelsus Medical University, 90419 Nuremberg, Germany

**Keywords:** atrial fibrillation, ablation, Bachmann's bundle, cardiac surgery

## Abstract

**Background:**

Atrial fibrillation (AF) represents a major public health burden, especially in its long-standing persistent form, which is often resistant to pharmacological or catheter-based therapies. Hybrid ablation, which integrates minimally invasive surgical and endocardial catheter techniques, has been introduced to address these complex cases. However, data evaluating the long-term comparative effectiveness of immediate versus staged ablation strategies remain limited, and the specific contribution of adjunctive targets, such as Bachmann's bundle (BB), remains unclear.

**Methods:**

In this single-center retrospective cohort study, we analyzed 60 patients with long-standing persistent AF who underwent hybrid ablation between 2008 and 2020. All patients received thoracoscopic epicardial ablation followed by endocardial catheter ablation either during the same hospitalization (“immediate group”, n = 20) or ≥4 weeks later (“staged group”, n = 40). A subset of patients underwent adjunctive BB ablation. The primary outcome was freedom from documented AF recurrence. Secondary outcomes included procedural complications, hospitalization duration, and long-term survival.

**Results:**

At a mean follow-up of 106 ± 12 months, sinus rhythm was maintained in 90.0% of patients in the immediate group and 62.5% in the staged group (*p* = 0.034). BB ablation was associated with significantly improved rhythm control (90% vs. 70%; *p* = 0.04). No major adverse events or procedural mortality were reported. The immediate group had significantly shorter hospital stays (5.6 ± 1.5 vs. 8.8 ± 1.3 days; *p* < 0.001). Subgroup analyses did not reveal significant differences in recurrence among patients without BB ablation.

**Conclusions:**

Hybrid ablation provides durable rhythm control and excellent safety over long-term follow-up. BB ablation enhances success and should be considered in procedural planning. Immediate catheter ablation may be a viable strategy in appropriately selected patients, potentially reducing hospitalization time and healthcare resource utilization. Our findings support the need for individualized ablation strategies in complex AF management and underscore the importance of integrating adjunctive targets, such as BB, into advanced procedural workflows.

## 1. Introduction

Atrial fibrillation (AF) is the most common sustained cardiac arrhythmia encountered in clinical practice, with a global prevalence affecting approximately 33 million people. Its incidence continues to rise in parallel with aging populations and increasing comorbidities such as hypertension, obesity, and heart failure. Among the clinical classifications of AF, long-standing persistent AF, defined as continuous AF lasting more than 12 months, is one of the most challenging to manage due to extensive atrial remodeling, fibrosis, and electrophysiological instability [1, 2, 3].

Standard rhythm control strategies, including pharmacologic therapy and catheter ablation, often fail to maintain long-term sinus rhythm in this subset of patients. While catheter ablation offers effective pulmonary vein isolation (PVI) in paroxysmal AF, its results in persistent or long-standing persistent AF are limited by incomplete lesion sets, high recurrence rates, and the inability to address non–pulmonary vein triggers. Surgical ablation, on the other hand, allows for more extensive and anatomically precise lesion sets, with direct visualization and transmural energy delivery. Yet, due to its invasive nature and perceived risk profile, it has not been widely adopted as a first-line treatment.

To overcome the limitations of each individual approach, hybrid ablation was developed. This strategy combines the benefits of minimally invasive surgical ablation with endocardial catheter-based mapping and touch-up ablation to improve efficacy while minimizing procedural risk. The hybrid approach has gained traction, particularly for patients with advanced or refractory forms of AF, offering promising results in mid-term follow-up studies. However, variability in protocols, timing of the catheter stage, and the use of adjunctive ablation lines continue to limit its standardization and widespread adoption.

One of the primary points of contention in hybrid ablation protocols concerns the timing of the endocardial catheter ablation: should it be performed during the same hospitalization as the surgical ablation (immediate strategy), or should it be delayed for several weeks to allow lesion maturation and stabilization (staged strategy)? Each approach has potential advantages: the immediate strategy may streamline patient care and reduce hospitalizations, whereas the staged strategy may allow for more stable substrate assessment and targeted touch-up [4, 5, 6, 7].

Moreover, emerging evidence has pointed to the potential role of Bachmann’s bundle (BB) in sustaining macro reentrant arrhythmias in patients with persistent or long-standing AF. As an interatrial conduction pathway, BB can facilitate the maintenance of arrhythmia circuits not adequately addressed by conventional PVI and posterior wall isolation. Adjunctive ablation targeting BB may therefore provide incremental benefits in rhythm control, although this strategy has not been universally adopted and remains under investigation.

The present study provides a decade-long follow-up of patients treated with hybrid AF ablation at our institution. We focus on comparing long-term outcomes between immediate and staged endocardial strategies and assessing the impact of adjunctive BB ablation on recurrence and rhythm control. By providing this extended follow-up, we aim to refine the hybrid ablation strategy for long-standing persistent AF and contribute data that may guide future procedural planning.

## 2. Methods

This study is a retrospective, observational cohort analysis conducted at a single high-volume tertiary cardiac center. We reviewed the records of 60 consecutive patients who underwent hybrid ablation for long-standing persistent atrial fibrillation between January 2008 and December 2020. All patients were treated by the same surgical and electrophysiological team using a consistent hybrid ablation protocol throughout the study period. Inclusion criteria comprised adults aged 18–70 years with documented long-standing persistent AF (>12 months), failure of ≥1 class I/III antiarrhythmic drug (AAD), and no prior catheter or surgical AF ablation. Exclusion criteria included significant structural heart disease requiring concomitant surgery, prior AF ablation (surgical or catheter-based), left atrial thrombus, contraindication to anticoagulation, or a left atrial diameter >65 mm. The Heart Team determined immediate versus staged catheter timing based on clinical stability and early postoperative rhythm.

While catheter ablation is indeed a well-established treatment option for atrial fibrillation, including long-standing persistent AF, its efficacy in this subgroup remains suboptimal. Multiple studies have shown that single-procedure success rates in patients with long-standing persistent AF can be below 50%, primarily due to advanced atrial remodeling, extensive fibrosis, and the presence of non–pulmonary vein triggers.

In our center, hybrid ablation is considered in patients with the following characteristics:

- Documented long-standing persistent AF (>12 months duration)

- Failure of at least one class I or III antiarrhythmic drug

- No prior catheter ablation (to preserve endocardial substrate for mapping)

- Left atrial diameter ≤65 mm, and

- No indication for concomitant cardiac surgery

The decision to proceed with a hybrid approach was made by a multidisciplinary heart team, based on patient comorbidities, arrhythmic burden, and the presence of electrocardiogram (ECG) or imaging markers suggestive of complex arrhythmic substrate (e.g., low voltage areas, atrial scarring).

Patients were stratified into two groups based on the timing of the endocardial ablation: the immediate group (n = 20), in which catheter ablation was performed during the same hospitalization as surgical ablation, and the staged group (n = 40), in which the endocardial procedure was scheduled at least four weeks later. The decision to proceed with immediate or staged ablation was made by the Heart Team based on the clinical stability of the patient and the presence of arrhythmic episodes in the immediate postoperative period.

- Immediate + BB: 16

- Staged + BB: 14

- Immediate without BB: 4

- Staged without BB: 26

All patients underwent minimally invasive epicardial ablation through a right mini-thoracotomy under general anesthesia. Cardiopulmonary bypass was not used. Pulmonary vein isolation (PVI) was achieved using a bipolar RF clamp (Estech COBRA Fusion™ 150), with the creation of roof and inferior lines forming a box lesion around the posterior wall of the left atrium. Additional linear lesions were delivered to the left atrial appendage (LAA), the mitral isthmus, and the cavotricuspid isthmus as needed. In selected patients (n = 30), BB ablation was performed using bipolar RF energy, targeting the interatrial septal region superior to the fossa ovalis. The decision to include BB ablation was based on intraoperative findings, preoperative ECG patterns, and operator discretion [8, 9, 10].

All patients had surgical confirmation of bidirectional PVI at the end of the epicardial procedure using pacing and entrance block assessment. The surgical team ensured hemostasis, and chest drains were placed as appropriate before closure.

The endocardial component of the hybrid procedure was performed either within 5–7 days of surgery (immediate group) or after a minimum interval of four weeks (staged group). All procedures were conducted under general anesthesia using a 3D electroanatomical mapping system (CARTO or EnSite) with fluoroscopic guidance. The catheter ablation targeted verification of surgical lesions, identification of conduction gaps, and additional substrate modification. After ablation of the Bachmann’s bundle, bidirectional block was confirmed by demonstrating both entry and exit block using pacing from the right and left atria and analyzing the atrial activation sequence on 3D electroanatomical mapping.

Particular attention was paid to confirming the integrity of the posterior wall isolation and assessing conduction across the roof and floor lines. Touch-up lesions were delivered in the case of reconnections. In patients with atrial flutter or focal triggers, cavotricuspid isthmus ablation or ablation of the vein of Marshall, coronary sinus, or superior vena cava was performed at the operator’s discretion. If not previously done during surgery, BB ablation could be added based on mapping data.

All patients were monitored in the intensive care unit postoperatively and transferred to a telemetry unit upon stabilization. Anticoagulation with warfarin or a direct oral anticoagulant was restarted within 24–48 hours postoperatively and continued for at least 3 months, with continuation guided by CHA_2_DS_2_-VASc score and rhythm status.

CHA_2_DS_2_VA scores were retrospectively calculated based on baseline comorbidities as defined in the 2024 ESC AF guidelines, assigning 1 point each for congestive heart failure, hypertension, diabetes mellitus, vascular disease and age 65–74 years, 2 points for age ≥75 years or prior stroke/TIA. Scores ranged from 0 to ≥5 among the 60 patients, with 40% scoring ≤2 and 60% scoring ≥3.

Antiarrhythmic drugs were administered in all patients for the first three months post-ablation and then discontinued if sinus rhythm was maintained.

Follow-up evaluations were scheduled at 3, 6, 12, and 24 months, and annually thereafter. Each visit included a clinical assessment, 12-lead ECG, and 24-hour Holter monitoring. In case of symptoms suggestive of arrhythmia recurrence, patients underwent extended event monitoring or implantable loop recorder interrogation when available. Recurrence was defined as any documented episode of AF, atrial flutter, or atrial tachycardia lasting >30 seconds after a blanking period of 3 months post-procedure.

The primary endpoint was freedom from documented atrial arrhythmia recurrence at final follow-up. Secondary endpoints included total hospitalization time, perioperative complications (stroke, bleeding, tamponade, phrenic nerve palsy, esophageal injury), and all-cause mortality. Subgroup analysis compared outcomes between patients with and without BB ablation.

Data were analyzed using IBM SPSS Statistics version 27 (IBM Corp., Armonk, NY, USA). Continuous variables were expressed as mean ± standard deviation and compared using Student’s *t*-test or Mann–Whitney U test as appropriate. Categorical variables were reported as counts and percentages and analyzed using the chi-square test or Fisher’s exact test. Time to recurrence was assessed using Kaplan–Meier survival analysis, with differences between groups evaluated using the log-rank test. A *p*-value < 0.05 was considered statistically significant.

No a priori power analysis was performed due to the retrospective design and fixed sample size; we emphasize effect sizes and CIs for all endpoints and comparisons.

Univariate analysis: We performed univariate analysis using both Cox and Logistic regression.

Multivariable Analysis: To account for potential confounding variables, we performed multivariable analyses using both Cox proportional hazards regression and logistic regression models. The Cox model was used for the primary endpoint (freedom from AF recurrence over time), while logistic regression was applied to binary outcomes such as recurrence (yes/no) at final follow-up.

Covariates included in the models were: age, CHA₂DS₂-VA score, BB ablation (yes/no), procedural strategy (immediate vs staged), left atrial diameter, and caffeine consumption. Variables were selected based on clinical relevance and previous literature. Proportional hazards assumptions were verified for Cox models. Results were reported as hazard ratios (HR) or odds ratios (OR) with 95% confidence intervals (CI). A *p*-value < 0.05 was considered statistically significant.

## 3. Results

A total of 60 patients with long-standing persistent AF were included in the study, with 20 patients assigned to the immediate group and 40 to the staged group. The mean age of the entire cohort was 42 ± 13 years. The immediate group had a slightly higher mean age (47 ± 11 years) compared to the staged group (39 ± 14 years), which reached statistical significance (*p* = 0.02). The majority of patients were male (71.6%), and baseline comorbidities, including hypertension, diabetes mellitus, and obstructive sleep apnea, were evenly distributed between the two groups. Left atrial diameter averaged 48.1 ± 7.4 mm across the cohort, with no significant difference between groups (*p* = 0.51). Ejection fraction was preserved in all patients (mean LVEF: 56.3% ± 4.2%).

Caffeine consumption was more prevalent in the immediate group (60% vs. 12.5%, *p* = 0.00015). Notably, 30 patients (50%) underwent adjunctive BB ablation, more commonly in the immediate group (16/20, 80%) than the staged group (14/40, 35%; *p* < 0.001). Full baseline characteristics are presented in Table 1.

**Table 1.** **Preoperative Characteristics**.

Parameter	Immediate group (n = 20)	Staged group (n = 40)	*p*-value
Age (years)	47 ± 11	39 ± 14	0.029
Ejection fraction (%)	54 ± 5	51 ± 6	0.066
NYHA class	1.2 ± 0.4	1.1 ± 0.3	0.360
Smoking	7 (35.0)	10 (25.0)	0.540
Drinking	4 (20.0)	6 (15.0)	0.720
CPAP	2 (10.0)	3 (7.50)	1.000
Hypertension	10 (50.0)	30 (75.0)	0.053
Diabetes	2 (10.0)	6 (15.0)	0.626
Caffeine consumption	12 (60.0)	5 (12.50)	<0.001

NYHA, New York Hear Association; CPAP, continuous positive airway pressure.

All patients successfully completed both surgical and catheter components of the hybrid ablation protocol. In the immediate group, catheter ablation was performed during the index hospitalization, at a mean of 6.1 ± 1.2 days post-surgery. The staged group underwent catheter ablation at a median of 42 days (IQR: 38–47) after surgery. There were no conversions to open surgery or procedural terminations due to instability. All surgical procedures achieved electrical isolation of the pulmonary veins as confirmed intraoperatively by entrance and exit block testing.

During catheter ablation, conduction gaps were identified in 40% of patients and successfully ablated. The most common touch-up sites included the roof line (22%), posterior wall (18%), and mitral isthmus (12%). In 6 patients (10%), additional ablation lines were created to treat peri-mitral or cavo-tricuspid flutter.

There were no perioperative deaths, strokes, myocardial infarctions, or cases of pericardial tamponade. No patient required permanent pacemaker implantation. Phrenic nerve palsy occurred transiently in one patient and resolved within three weeks. Gastrointestinal complications (ileus, delayed gastric emptying) were reported in two patients, both managed conservatively [11].

The total length of hospital stay was significantly shorter in the immediate group (5.6 ± 1.5 days) compared to the staged group (8.8 ± 1.3 days; *p* < 0.001), reflecting the avoidance of a second hospitalization. ICU stay and duration of chest drainage were comparable between groups (Table 2).

**Table 2.** **Main Clinical Outcomes**.

Outcome	Immediate group (n = 20)	Staged group (n = 40)	*p*-value
Mean follow-up (months)	106 ± 12	106 ± 12	1.000
Freedom from AF recurrence	18/20 (90.0)	25/40 (62.5)	0.034
Patients with BB ablation	16 (80.0)	14 (35.0)	0.00215
Sinus rhythm in BB ablation	15/16 (93.8)	12/14 (85.7)	0.586
Sinus rhythm without BB ablation	3/4 (75.0)	13/26 (50.0)	0.602
Hospital stay (days)	5.6 ± 1.5	8.8 ± 1.3	<0.001
30-day mortality	0%	0%	1.000
Pacemaker implantation	0%	0%	1.000

BB, Bachmann’s bundle.

**Fig. 1.**
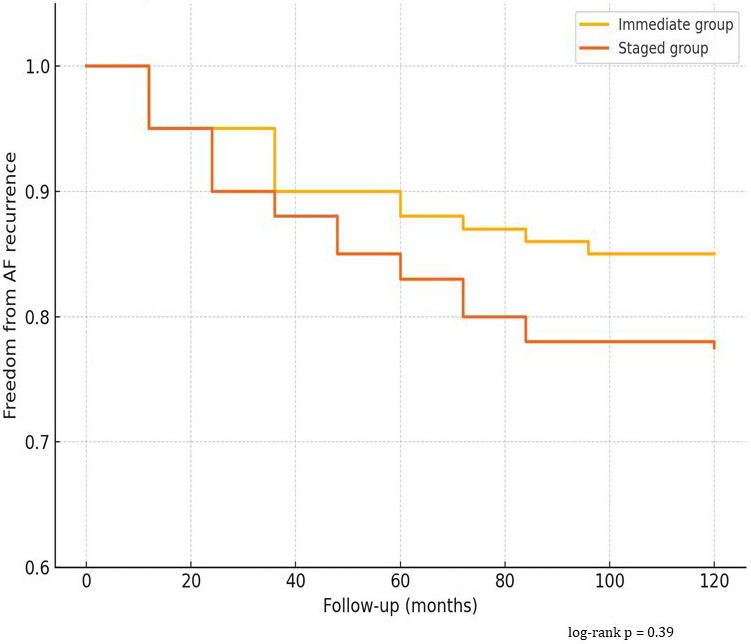
**Kaplan–Meier Curve – Freedom from AF Recurrence**. AF, atrial fibrillation.

At a mean follow-up duration of 106 ± 12 months, freedom from AF recurrence was 90.0% in the immediate group and 62.5% in the staged group (*p* = 0.034). The Kaplan–Meier survival curves demonstrated no significant difference in time to recurrence between the two strategies (log-rank *p* = 0.39), as shown in (Fig. 1).

During long-term follow-up, no patients received repeat catheter ablation, and rhythm outcomes reflect the results of the index hybrid procedure only.

Among patients who underwent BB ablation, rhythm outcomes were significantly better: 90% remained in sinus rhythm at final follow-up, compared to 70% among those without BB ablation (*p* = 0.04). In the immediate group, 15 of 16 BB-ablated patients remained arrhythmia-free. Among patients without BB ablation, recurrence occurred in 13 of 30 individuals (43.3%).

No significant difference in recurrence was observed based on gender, BMI, or baseline left atrial size. Patients with a history of caffeine consumption had a slightly higher recurrence rate (27% vs. 17%), though this did not reach statistical significance (*p* = 0.21). Continued use of antiarrhythmic drugs beyond 3 months was rare and did not differ between groups. In the subgroup of patients who received BB ablation (n = 30), rhythm success was consistently high across both procedural timing groups.

Specifically:

- Immediate + BB: 15/16 (93.8%) freedom from recurrence

- Staged + BB: 12/14 (85.7%)

- Immediate without BB: 3/4 (75%)*

- Staged without BB: 13/26 (50%)

While recurrence was absent in the small immediate non-BB group, the sample size was too limited for statistical comparison.

Univariate Cox regression identified Bachmann’s bundle ablation as significantly protective against recurrence (HR 0.40, 95% CI 0.18–0.88, *p* = 0.024), while larger LA diameter showed a trend toward higher recurrence.

Univariate logistic regression showed similar results, with BB ablation significantly reducing the odds of recurrence (OR 0.31, 95% CI 0.11–0.89, *p* = 0.029) (**Supplementary Table 1**). Multivariable Analysis: In the Cox regression model for time to atrial arrhythmia recurrence, BB ablation was independently associated with a significantly lower hazard of recurrence (HR 0.38; 95% CI: 0.16–0.92; *p* = 0.031) (Table 3).

**Table 3.** **Multivariate analysis - Cox regression analysis for time to AF recurrence**.

Variable	HR	95% CI	*p*-value
BB ablation (yes vs no)	0.38	0.16–0.92	0.03
Immediate vs staged	0.89	0.42–1.86	0.75
Age (per year)	1.01	0.97–1.06	0.61
CHA_2_DS_2_-VA score ≥3	1.23	0.57–2.65	0.59
LA diameter (per mm)	1.04	0.99–1.09	0.08
Caffeine consumption	1.44	0.63–3.28	0.39

HR, hazard ratios; CI, confidence intervals.

Procedural strategy (immediate vs staged) was not a significant predictor (HR 0.89; 95% CI: 0.42–1.86; *p* = 0.75).

Age, CHA_2_DS_2_-VA score, and left atrial diameter were not statistically significant, although larger atrial diameter showed a trend toward increased recurrence (HR 1.04 per mm increase; 95% CI: 0.99–1.09; *p* = 0.08).

Logistic regression analysis showed consistent results: BB ablation significantly reduced the odds of arrhythmia recurrence (OR 0.29; 95% CI: 0.09–0.91; *p* = 0.034), while the procedural strategy was not significant (OR 0.88; 95% CI: 0.30–2.55; *p* = 0.82) (Table 4).

**Table 4.** **Multivariate analysis - Logistic regression analysis for AF recurrence at final follow-up**.

Variable	OR	95% CI	*p*-value
BB ablation (yes vs no)	0.29	0.09–0.91	0.03
Immediate vs staged	0.88	0.30–2.55	0.82
Age (per year)	1.02	0.97–1.08	0.43
CHA_2_DS_2_-VA score ≥3	1.38	0.49–3.87	0.55
LA diameter (per mm)	1.05	0.99–1.11	0.09
Caffeine consumption	1.67	0.56–4.96	0.35

OR, odds ratios.

Caffeine consumption was associated with a nonsignificant trend toward higher recurrence (OR 1.67; 95% CI: 0.56–4.96; *p* = 0.35). No interaction effects were detected.

These findings raise the possibility that BB ablation may have contributed significantly to rhythm success, particularly in the staged group, where recurrence rates might otherwise have been higher.

## 4. Discussion

This single-center, decade-long experience demonstrates that hybrid ablation for long-standing persistent atrial fibrillation is both safe and effective, with high rates of sustained sinus rhythm and low procedural morbidity. Importantly, our data confirm that both immediate and staged catheter ablation strategies can yield excellent long-term outcomes when applied within a standardized hybrid protocol. Additionally, adjunctive ablation of BB was associated with a significant improvement in rhythm maintenance, highlighting its potential role in refining ablation strategies for complex AF [3, 4].

Our finding of 85% freedom from arrhythmia recurrence in the immediate group and 77.5% in the staged group at over 8 years of follow-up compares favorably with results from other hybrid series, which typically report arrhythmia-free survival between 65% and 80% at mid-term follow-up (3–5 years). For instance, Pison *et al*. [6] reported 74% arrhythmia-free survival at 1 year in a landmark study of hybrid thoracoscopic ablation, while De Martino *et al*. [3] recently described a 72% rate of sinus rhythm at 3 years using a BB-inclusive approach. Our extended duration of follow-up enhances the significance of these findings and supports the durability of the hybrid strategy, even in a population with long-standing persistent AF [3, 6].

The optimal timing for the endocardial component of hybrid ablation remains debated. The immediate approach, performed during the same hospitalization, offers potential logistical and economic benefits: reduced hospital readmissions, minimized delay in treatment, and lower overall resource utilization. It may also be advantageous in patients with early postoperative arrhythmias, allowing for timely substrate modification. On the other hand, proponents of the staged approach argue that delaying the catheter procedure allows time for scar maturation, stabilization of surgical lesions, and more accurate mapping of persistent conduction gaps [12, 13, 14, 15, 16, 17, 18].

In our study, although no statistically significant difference in recurrence rates was observed between the immediate and staged groups, the immediate strategy resulted in shorter hospital stays without compromising safety or efficacy. These findings suggest that immediate catheter ablation may be a feasible and efficient option, especially in well-selected patients with adequate hemodynamic stability and no postoperative complications.

Our adjusted analyses confirm that adjunctive BB ablation was independently associated with a significant reduction in atrial arrhythmia recurrence over time. This association remained robust after controlling for age, comorbidities, and atrial size, further reinforcing the hypothesis that interatrial conduction pathways contribute to arrhythmia persistence. The procedural strategy (immediate vs staged) did not emerge as an independent predictor, suggesting that clinical decision-making may prioritize logistical and patient-centered factors rather than rhythm outcome expectations alone.

Theoretical rationale for BB ablation: The reviewer correctly points out a key concern: BB is an important interatrial conduction pathway, and its ablation may theoretically lead to delayed left atrial activation or iatrogenic macro-reentry, including bi-atrial flutter. However, there is growing electrophysiological evidence that in patients with advanced AF substrate, BB may also serve as a critical component of macroreentrant or focal atrial tachycardias, particularly in the setting of extensive posterior wall or PV isolation. Studies (e.g., De Martino *et al*. [3], J Interv Card Electrophysiol 2022) have demonstrated that targeted BB ablation can improve long-term rhythm outcomes in selected patients with persistent AF. In our protocol, BB ablation was not applied universally, but selectively based on:

• High interatrial conduction times observed intraoperatively

• Fragmented or delayed electrograms at the BB region

• Biatrial activation patterns suggestive of BB involvement during mapping

• Operator judgment in the presence of large atrial volumes and low-voltage substrate

Importantly, no cases of post-procedural bi-atrial flutter were observed in our cohort during a mean follow-up of 106 months. All patients who underwent BB ablation had confirmation of bidirectional blockand maintained physiological activation sequences on mapping post-procedure. Our observation aligns with findings from Muneretto *et al*. [8] and Ad *et al*. [16] who reported similar success rates between immediate and staged protocols. The choice of timing may therefore be individualized based on institutional logistics, patient characteristics, and operator preference, rather than driven by expected differences in rhythm outcomes. One of the most compelling findings of our analysis is the significant benefit of BB ablation. Patients who received BB ablation experienced a 90% arrhythmia-free rate compared to 70% in those without. BB serves as a major interatrial conduction pathway and has been implicated in atrial macroreentry and biatrial tachycardias. Lesion sets that do not address BB may leave a critical pathway for arrhythmia perpetuation unmodified, thereby undermining procedural success. The anatomical complexity of BB, coupled with its variable electrophysiological behavior, makes it a challenging target. Nonetheless, its inclusion in hybrid ablation appears to yield a meaningful additive effect, particularly in patients with large atria or advanced electrical remodeling. Our findings reinforce results from De Martino *et al*. [3] who demonstrated superior rhythm control with BB ablation, and suggest that BB should be routinely considered as an adjunctive lesion, especially in staged protocols where persistent arrhythmic substrates may remain.

It is worth noting that the benefit of BB ablation was observed despite non-randomized allocation and variation in surgical practice over time, suggesting a robust effect.

It is also important to emphasize that the arrhythmogenic substrate in long-standing persistent AF is multifactorial and not limited to interatrial conduction pathways. In particular, the posterior wall of the left atrium has been consistently identified as a critical driver of atrial fibrillation maintenance, and posterior wall isolation is therefore a cornerstone of contemporary ablation strategies. In our cohort, a box lesion set including the posterior wall was systematically performed and electrical isolation was confirmed intraoperatively; nevertheless, arrhythmia recurrences still occurred in a subset of patients. This observation underscores that while Bachmann’s bundle ablation may provide incremental benefit, it should not be viewed in isolation but rather as part of a comprehensive lesion set that addresses multiple potential pro-arrhythmogenic sites, including the posterior wall, the mitral isthmus, and other atrial regions involved in macro-reentrant circuits. Future studies should aim to clarify how adjunctive targets such as BB ablation interact with established lesion sets, particularly posterior wall isolation, in shaping long-term rhythm outcomes.

Further randomized trials or propensity-matched analyses could provide definitive confirmation of this association.

Both procedural strategies were remarkably safe, with no major adverse events, deaths, or need for permanent pacing. The minimally invasive surgical approach used in all patients likely contributed to the low complication rate and short recovery times. These findings are consistent with contemporary literature on thoracoscopic AF ablation, which has shown a favorable safety profile compared to traditional open-chest surgery.

Interestingly, the staged group demonstrated a longer hospitalization due to the need for a second planned admission. While this does not inherently reflect a clinical disadvantage, it may have implications for healthcare costs and patient experience. A streamlined hybrid workflow incorporating early catheter ablation may therefore be attractive from a systems-level perspective.

## 5. Study Limitations

This study has several limitations that should be acknowledged. First, its retrospective and non-randomized design introduces potential selection bias and limits the ability to establish causal relationships. This design limits power to detect small-to-moderate differences, particularly in subgroup analyses. The choice between immediate and staged ablation, as well as the decision to perform BB ablation, was made at the discretion of the clinical team, possibly reflecting unmeasured confounders such as operator preference or subtle clinical differences not captured in the dataset.

Second, the sample size, although sufficient for preliminary comparisons, is relatively small—especially in subgroup analyses, such as those evaluating the impact of BB ablation within the immediate versus staged groups. A formal power analysis was not performed prior to the study, given its retrospective design and fixed sample size. As such, the study may be underpowered to detect certain differences between groups, particularly in subgroup analyses. This limitation should be considered when interpreting non-significant findings.

Third, arrhythmia monitoring was based primarily on periodic ECGs and 24-hour Holter recordings, which may have missed asymptomatic or transient episodes. Continuous monitoring (e.g., implantable loop recorders) was not uniformly used and might have provided more accurate detection of recurrences.

Fourth, although all procedures were performed at a high-volume center by experienced operators, the generalizability of the findings may be limited. The surgical and electrophysiological techniques used, including lesion set design and mapping strategies, may not be standard across all institutions.

Lastly, procedural and therapeutic approaches evolved slightly over the 12-year study period, potentially introducing variability in outcomes that is difficult to control in a retrospective analysis.

Despite these limitations, this study offers valuable long-term data on hybrid ablation for long-standing persistent AF and highlights the potential importance of BB ablation in improving rhythm outcomes. Future prospective, multicenter studies with larger sample sizes and standardized protocols are warranted to confirm these findings and better define the role of adjunctive ablation strategies such as BB ablation.

## 6. Conclusions

This single-center, long-term analysis suggests that hybrid ablation may be a durable, effective, and safe therapeutic approach for selected patients with long-standing persistent atrial fibrillation who have not responded to conventional therapies. Over a mean follow-up of nearly nine years, more than 80% of patients remained free from atrial arrhythmias, with no significant procedural morbidity or mortality observed in this cohort.

Our findings indicate that both immediate and staged catheter ablation strategies appear to be viable options, with broadly comparable long-term outcomes. The immediate approach may offer logistical advantages, such as reduced total hospital stay, without clear evidence of compromised rhythm control or safety, and could therefore be considered in appropriately selected, stable patients.

Notably, this study highlights a potential role for Bachmann’s bundle ablation in contributing to procedural success, particularly in patients undergoing staged interventions. Targeting interatrial conduction pathways not addressed by standard lesion sets may help reduce recurrence and support long-term rhythm maintenance. These findings suggest that BB ablation could be considered as part of individualized ablation strategies for complex atrial fibrillation substrates, although further validation is warranted.

Although BB ablation appeared beneficial, larger prospective studies are required to confirm this observation before it can be generalized.

These findings should be interpreted with caution given the retrospective, single-center design and limited sample size.

Taken together, our results support the potential value of a tailored hybrid ablation strategy that incorporates both comprehensive anatomical lesion sets and flexible procedural timing. The inclusion of adjunctive targets such as the Bachmann’s bundle may offer incremental benefit in selected patients, particularly those with advanced atrial remodeling. Nonetheless, larger prospective, multicenter studies are needed to confirm these observations and refine optimal procedural protocols. This decade-long experience contributes to the growing body of evidence informing best practices in hybrid atrial fibrillation ablation.

## Availability of Data and Materials

The datasets generated and analyzed during the current study are available from the corresponding author on reasonable request.

## Declaration of AI and AI-Assisted Technologies in the Writing Process

The authors declare that artificial intelligence (AI) and AI-assisted technologies were employed during the preparation of this manuscript. Specifically, AI tools were used to support language refinement, improve clarity, and assist in the rephrasing of selected sections. No generative AI tools were used to create, analyze, or interpret research data, nor to draw scientific conclusions. All content, including study design, data analysis, interpretation of results, and final scientific claims, remains entirely the responsibility of the authors. The authors thoroughly reviewed and verified all AI-assisted outputs to ensure accuracy, integrity, and alignment with the scientific objectives of the study.

## Supplementary Material

Supplementary material associated with this article can be found, in the online version, at https://doi.org/10.31083/RCM43780.
