# Supplementary material for: Hybrid Atrial Fibrillation Ablation: A Decade-Long Single-Center Experience
Source: Rev Cardiovasc Med. 2025 Dec 23;26(12):43780. doi: 10.31083/RCM43780 (PMC12781017; doi:10.31083/RCM43780)
Supplement: Supplementary file 1 [file 2153-8174-26-12-43780-s1.docx]

Supplementary Table 1. Univariate regression analyses for predictors of AF recurrence.

| Variable | Univariate Cox regression HR (95% CI) | *p*-value | Univariate Logistic regression OR (95% CI) | *p*-value |
| --- | --- | --- | --- | --- |
| Bachmann’s bundle ablation (yes vs no) | 0.40 (0.18–0.88) | 0.024 | 0.31 (0.11–0.89) | 0.029 |
| Immediate vs staged strategy | 0.92 (0.46–1.83) | 0.81 | 0.91 (0.33–2.53) | 0.86 |
| Age (per year) | 1.01 (0.97–1.06) | 0.61 | 1.02 (0.97–1.08) | 0.43 |
| CHA₂DS₂-VA score ≥3 | 1.25 (0.60–2.60) | 0.55 | 1.36 (0.50–3.70) | 0.56 |
| Left atrial diameter (per mm) | 1.04 (0.99–1.09) | 0.07 | 1.05 (0.99–1.11) | 0.08 |
| Caffeine consumption (yes vs no) | 1.48 (0.65–3.35) | 0.35 | 1.64 (0.58–4.67) | 0.35 |
